# Supplementary material for: FKBP51 in glutamatergic forebrain neurons promotes early life stress inoculation in female mice
Source: Nat Commun. 2025 Mar 14;16:2529. doi: 10.1038/s41467-025-57952-x (PMC11912546; doi:10.1038/s41467-025-57952-x)
Supplement: Supplementary file 1 — Supplementary Information [file 41467_2025_57952_MOESM1_ESM.docx]

**Supplementary information**

van Doeselaar et al., 2024: FKBP51 in glutamatergic forebrain neurons promotes early life stress inoculation in female mice: Association with the transcriptional regulator TCF4

**This PDF file includes:**

Supplementary Table 1-2

Supplementary Figures 1-10

**Table S1.**

| **Name WGCNA subnetwork** | **No. of genes** | **Association** | **p-value** |
| --- | --- | --- | --- |
| Antiquewhite | 30 | ELS | < 0.01 |
| Black | 272 | Genotype x ELS | < 0.01 |
| Blue | 733 | ELS | < 0.001 |
| Cyan | 193 | Genotype | < 0.01 |
| Darkmagenta | 60 | Genotype | < 0.001 |
| Darkorange | 176 | ELS | < 0.01 |
| Darkorange | 176 | Genotype x ELS | < 0.001 |
| Green | 342 | ELS | < 0.001 |
| Greenyellow | 235 | Genotype | < 0.01 |
| Grey | 7878 | ELS | < 0.001 |
| Grey60 | 156 | ELS | < 0.001 |
| Lavenderblush | 34 | Genotype x ELS | < 0.01 |
| Lightgreen | 141 | ELS | < 0.01 |
| Lightyellow | 112 | Genotype | < 0.001 |
| Red | 293 | Genotype x ELS | < 0.01 |
| Royalblue | 108 | ELS | < 0.001 |
| Salmon | 196 | Genotype x ELS | < 0.001 |
| Skyblue | 84 | ELS | < 0.01 |
| Turquoise | 1074 | Genotype | < 0.001 |

**Table S1.** Overview of the significantly regulated WGCNA networks and their association with genotype, ELS, or genotype by ELS interaction. Statistical test: 2-way ANOVA.

**Table S2.**

| **Primer Name** | **Sequence** |
| --- | --- |
| *Slc17a6_*Fwd | TGGAAAATCCCTCGGACAGAT |
| *Slc17a6_*Rev | CATAGCGGAGCCTTCTTCTCA |
| *HPRT_*Fwd | ACCTCTCGAAGTGTTGGATACAGG |
| *HPRT_*Rev | CTTGCGCTCATCTTAGGCTTTG |
| *TCF4_*Fwd | CGAAAAGTTCCTCCGGGTTTG |
| *TCF4-*Rev | CGTAGCCGGGCTGATTCAT |
| *Foxp2-*Fwd | AGTGTGCCCAATGTGGGAG |
| *Foxp2_*Rev | CATGATAGCCTGCCTTATGAGTG |
| *Polr2b_*Fwd | GACGACGATGAGATCACTCCG |
| *Polr2b_*Rev | GGTGCATCTTCCACAATTCTTTG |
| *Plcb4_*Fwd | GGACAAGTGCTAGAATGTTCCC |
| *Plcb4_*Rev | GAAGCCGATATTCACCAGATCC |
| *Rab37_*Fwd | CCCTTCAGCCCGAACTACG |
| *Rab37*_Rev | AAGGCCCCGTCTTTGAATTGG |
| *Zic1*_Fwd | TGCGATAAGTCCTACACGCAC |
| *Zic1*_Rev | CGTGGACGACTCATACCCC |
| *Nr3c1*_Fwd | AGCTCCCCCTGGTAGAGAC |
| *Nr3c1*_Rev | GGTGAAGACGCAGAAACCTTG |
| *Plekhg1*_Fwd | GAGTGGGACACCACAAACCA |
| *Plekhg1*_Rev | TCCAGGTAACCCTCCACGAT |
| *Tcf7l2*_Fwd | AACGAACACAGCGAATGTTTCC |
| *Tcf7l2*_Rev | CTCGGCATTTCTTAGGAGCG |
| *Zic4*_Fwd | TGTACACCCGAGTGGGATGA |
| *Zic4*_Rev | GCCTGGAGTGGCACCTAAAA |

**Table S2.** Primers used for qPCR.

**
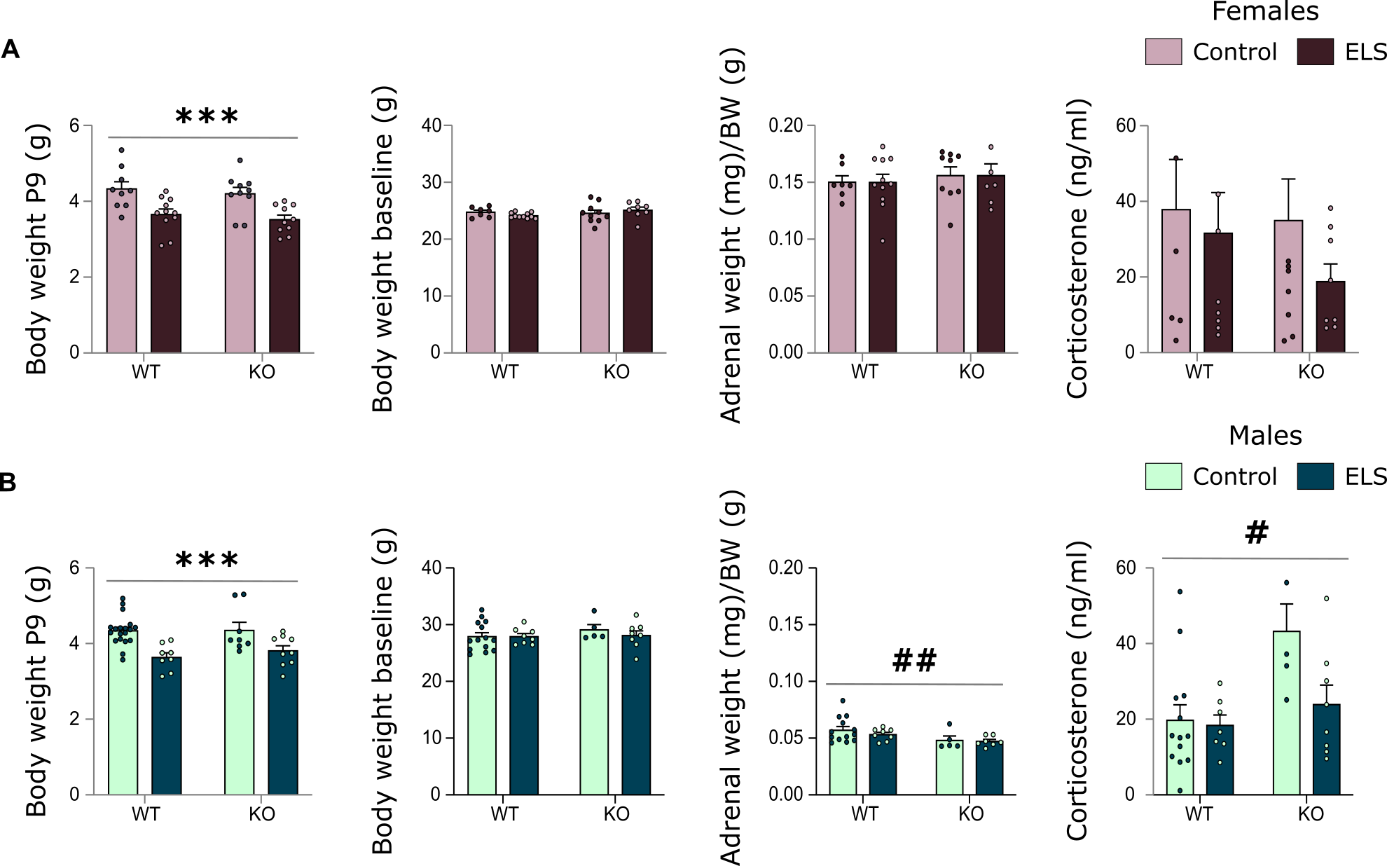
**

**Supplementary Figure 1. Differential physiological effects of early life stress exposure and loss of FKBP51 in glutamatergic forebrain neurons in male and female mice.**

Male and female offspring, including *Fkbp5^Nex^* and *Fkbp5^lox/lox^* mice, underwent a limited bedding and nesting (LBN) early life stress (ELS) exposure from postnatal day 2 (P2) to P9. In females (A) this led to a reduced body weight at the end of the procedure in both genotypes. In adulthood however, body weight at the start of the experiment and adrenal weight and baseline corticosterone concentrations remained unaffected by both ELS exposure and genotype. Male mice (B) also had reduced body weight as a result of ELS exposure at the end of LBN paradigm, but in adulthood body weight was unaffected. A main effect of genotype was however found in both adrenal weight and for baseline corticosterone levels. Error bars represent mean + S.E.M. All panels: 2-way ANOVA. *** effect of ELS p < 0.001, ^#^ effect of genotype p < 0.05; ^##^ effect of genotype p < 0.01

**
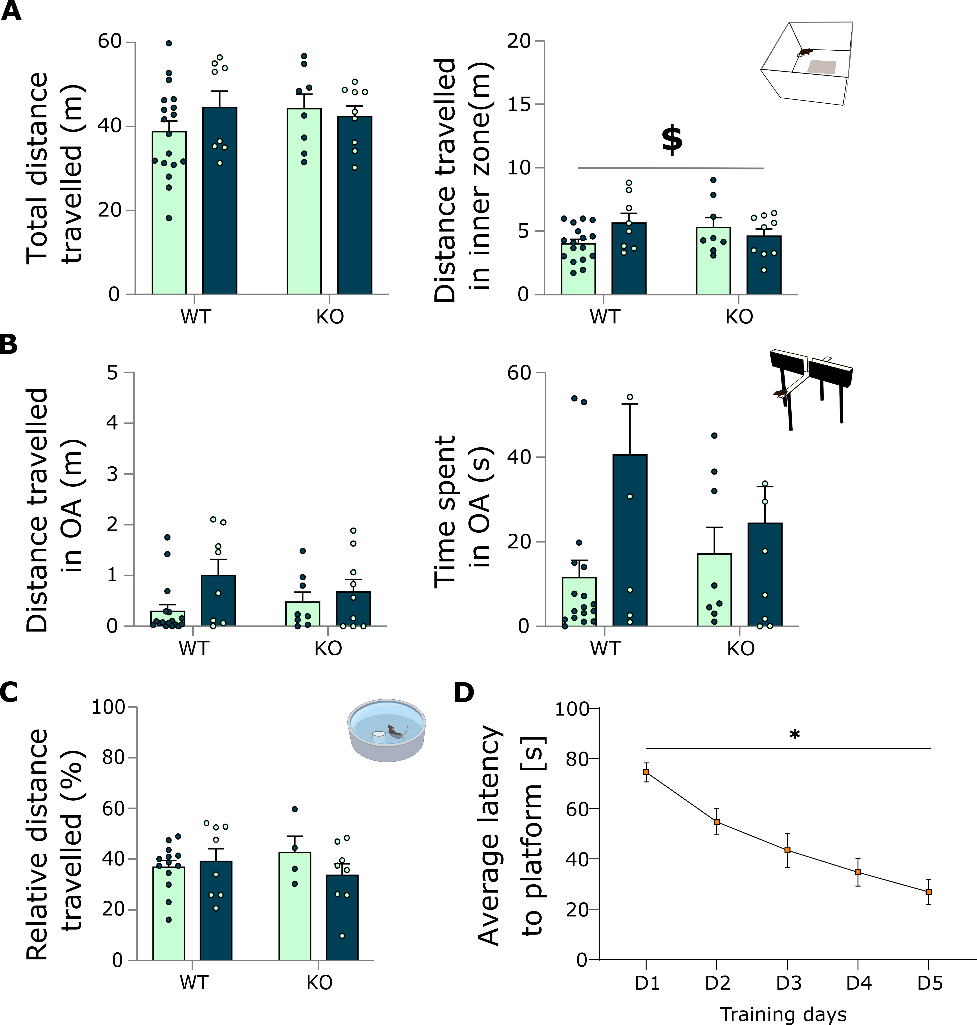
**

**Supplementary Figure 2**. **Effects of early life stress and loss of FKBP51 in glutamatergic forebrain neurons on anxiety-like behaviour and cognitive memory function in male mice.**

Male offspring, including *Fkbp5^Nex^* and *Fkbp5^lox/lox^* mice, underwent a limited bedding and nesting (LBN) early life stress (ELS) exposure from postnatal day 2 (P2) to P9 and were tested on a number of behavioral tests in adulthood. Locomotor behaviour of male mice remained unaffected (A), however an interaction effect between ELS exposure and *Fkbp5* genotype was found for distance travelled in the inner zone of the open field test (OF). However, (B) when further investigating anxiety-like behaviour in the elevated plus maze (EPM), no significant differences of ELS exposure and genotype or their interaction were found. Neither ELS exposure, nor genotype affected spatial memory performance under stressful environments (MWM) (C). Males cohort: ELS n = 9 *Fkbp5^Nex^* and n = 8 *Fkbp5^lox/lox^*; controls: n = 8 *Fkbp5^Nex^* and n = 18 *Fkbp5^lox/lox^*. (D) WT females significantly reduce the latency to find the hidden platform over the course of the training days, when the inter-trial interval is shortened to 10 minutes. N = 13. Error bars represent mean + S.E.M. Panles A-C: 2-way ANOVA. Panel D: repeated measures ANOVA. ^$^ interaction effect ELS x genotype p < 0.05. Image of MWM created in BioRender. Schmidt, M. (2025) <https://BioRender.com/o64z476>.

**
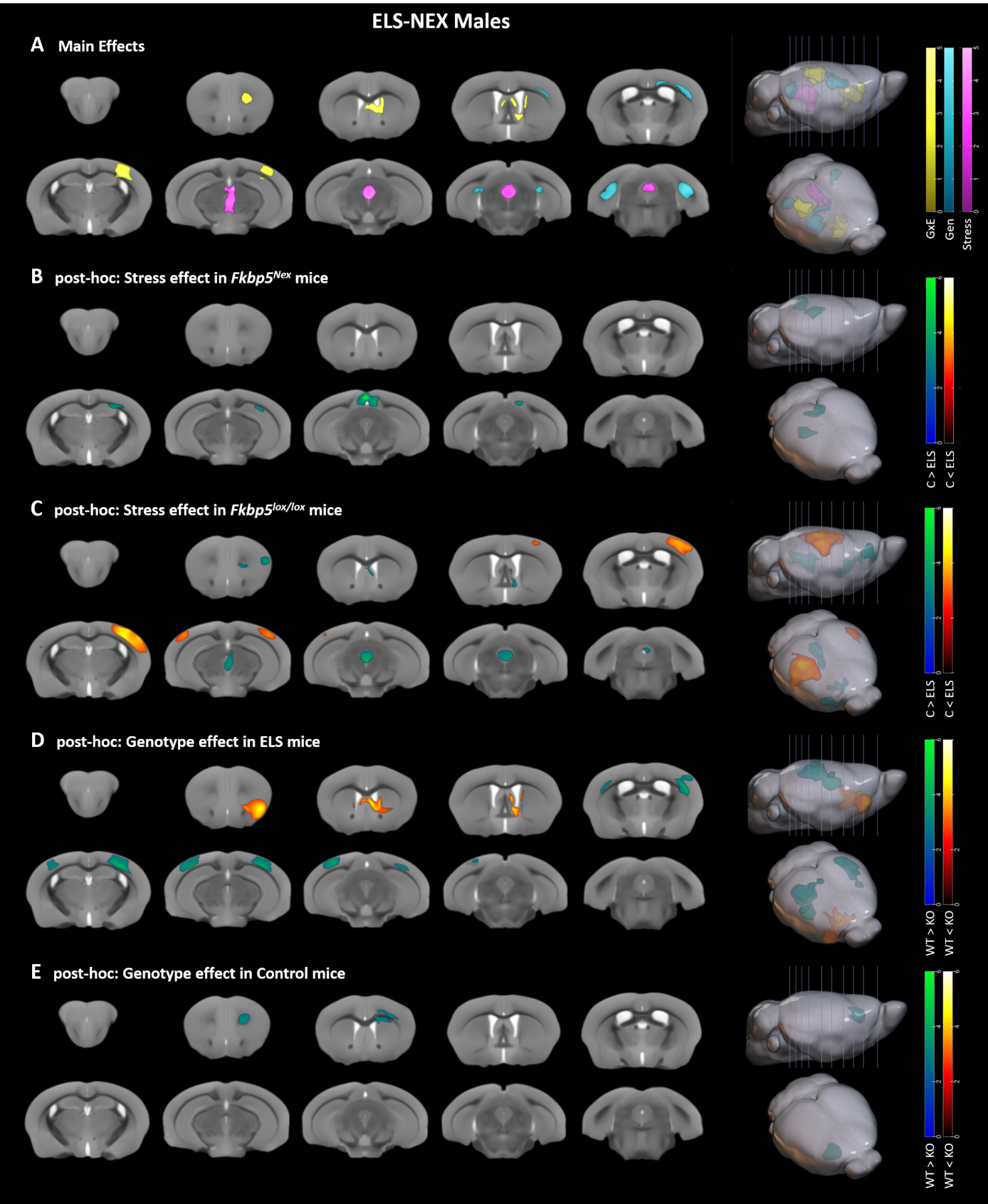
**

**Supplementary Figure 3. ELS and *Fkbp5* genotype lead to separate and interactive changes in brain volume in male mice.**

(A) Deformation-based morphometry analyses of the male brains revealed main effects of genotype in the bilateral subiculum. An effect of ELS was apparent in the periaqueductal gray (PAG), while an ELS x genotype interaction was observed for the right somatosensory cortex and the septal nucleus extending to the anterior forceps of the corpus callosum. (B) ELS exposure in *Fkbp5^Nex^* mice led to a reduced volume in the retrosplenial cortex and the right hippoxampus. (C) ELS exposure in *Fkbp5^lox/lox^* males resulted in a larger somatosensory cortex and a reduction in the volume of the PAG, right BNST and right somatosensory cortex. (D) Under ELS conditions, *Fkbp5^lox/lox^* mice had a larger somatosensory cortical volume compared to *Fkbp5^Nex^* mice, whereas the BNST and the septal nucleus were larger in *Fkbp5^Nex^* mice. (E) Unstressed control males had larger volumes in the right anterior forceps and the dorsal part of the corpus callosum compared to *Fkbp5^Nex^* mice. Scales represent Z-scores. WT = wild-type, KO = Fkbp5^Nex^, C = control, ELS = early life stress, Gen = genotype, GxE = gene by environment interaction. ELS: *Fkbp5^lox/lox^* n = 8, *Fkbp5^Nex^* n = 8; control: *Fkbp5^lox/lox^* n = 14 and *Fkbp5^Nex^* n = 5.

**
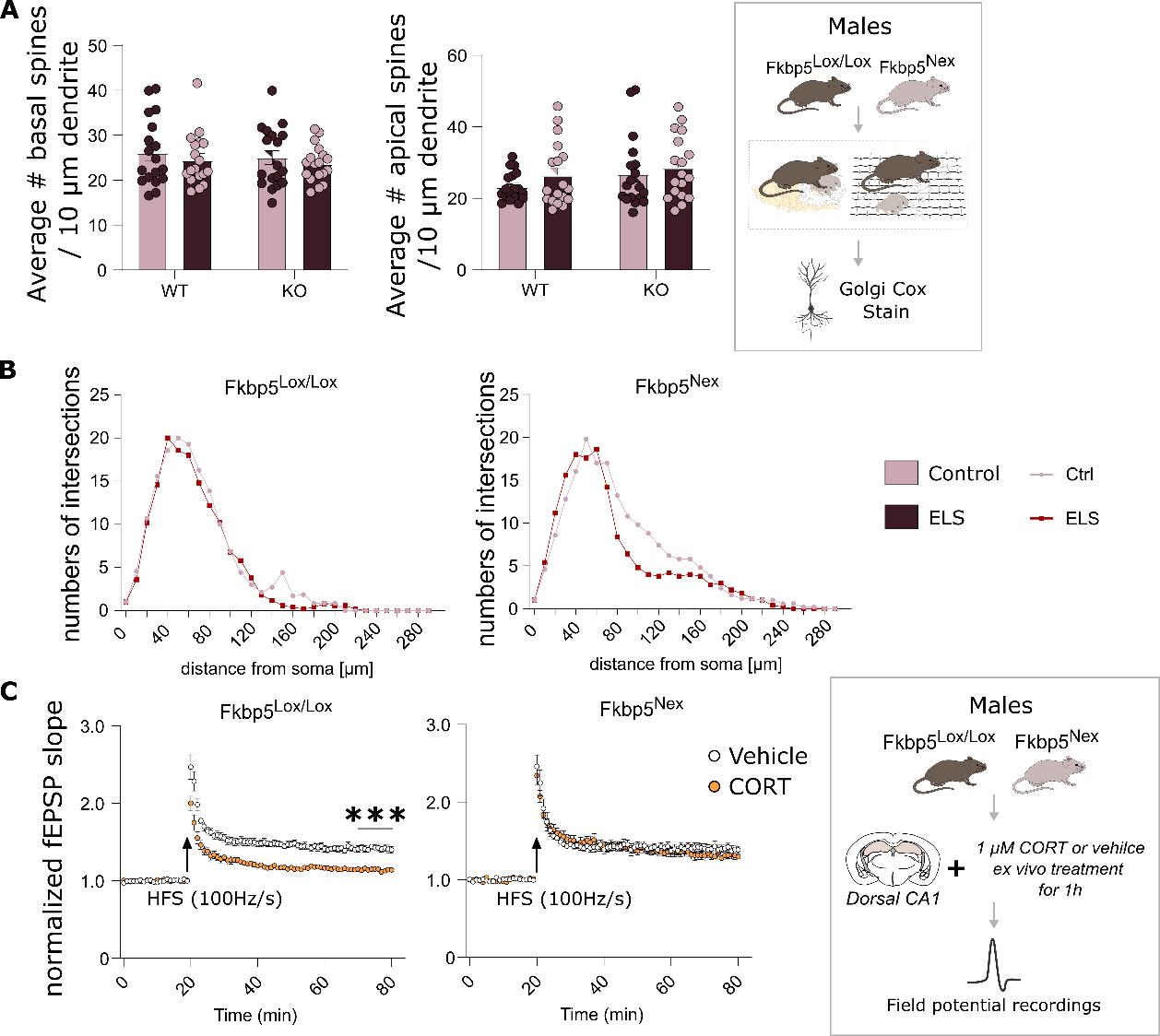
**

**Supplementary Figure 4. Neuronal structure and function in male FKBP5^Nex^ and control mice with or without ELS.**

A separate cohort of male *Fkbp5^Nex^* and *Fkbp5^lox/lox^* offspring was exposed to a limited bedding and nesting (LBN) early life stress (ELS) paradigm and a Golgi Cox staining was performed at the hippocampus of 8-month old male mice. No significant effects on spine density (A; n = 18 neurons for all groups) or dendritic complexity (B; n = 5-7 neurons per group) were observed. (C) Corticosterone exposure significantly decreased LTP in WT, but not FKBP5^Nex^ mice (n = 7 mice, n = 14 slices per condition). Error bars represent mean + S.E.M. Panel A: 2-way ANOVA. Panel B, C: repeated measures ANOVA. *** effect of CORT p < 0.001. Image of neuron created in BioRender. Schmidt, M. (2025) <https://BioRender.com/o64z476>.

**
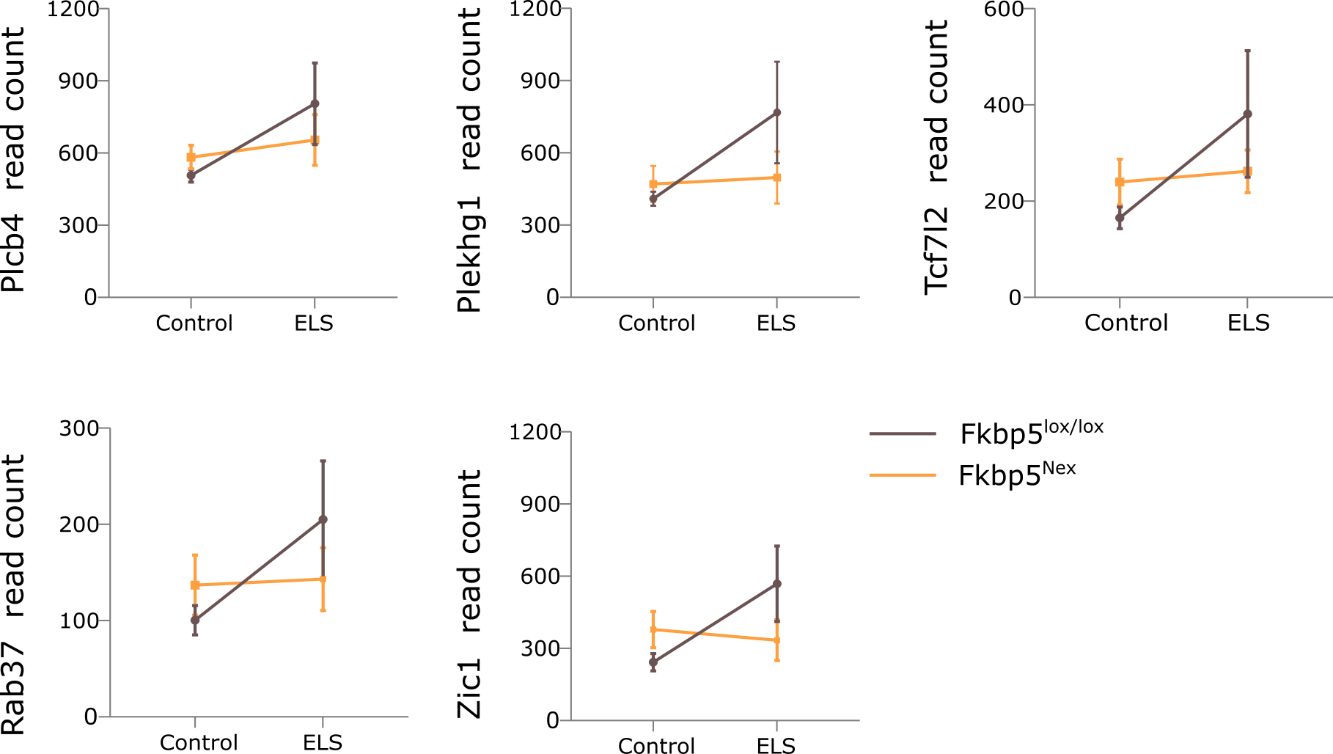
**

**Supplementary Figure 5. Hub genes of the darkorange network.**

The five hubgenes of the darkorange network from the WGCNA analyses, that was associated to early life stress (ELS) exposure and ELS x genotype interaction, showed a similar expression pattern in the hippocampus. Upon ELS, gene expression was upregulated in wild-type *Fkbp5^lox/lox^* animals, whereas gene expression in *Fkbp5^Nex^* animals was more stable following ELS. Each data point represents a distinct group of mice, and lines are used solely to enhance visual clarity. Error bars represent mean + S.E.M. All panels: 2-way ANOVA.

**
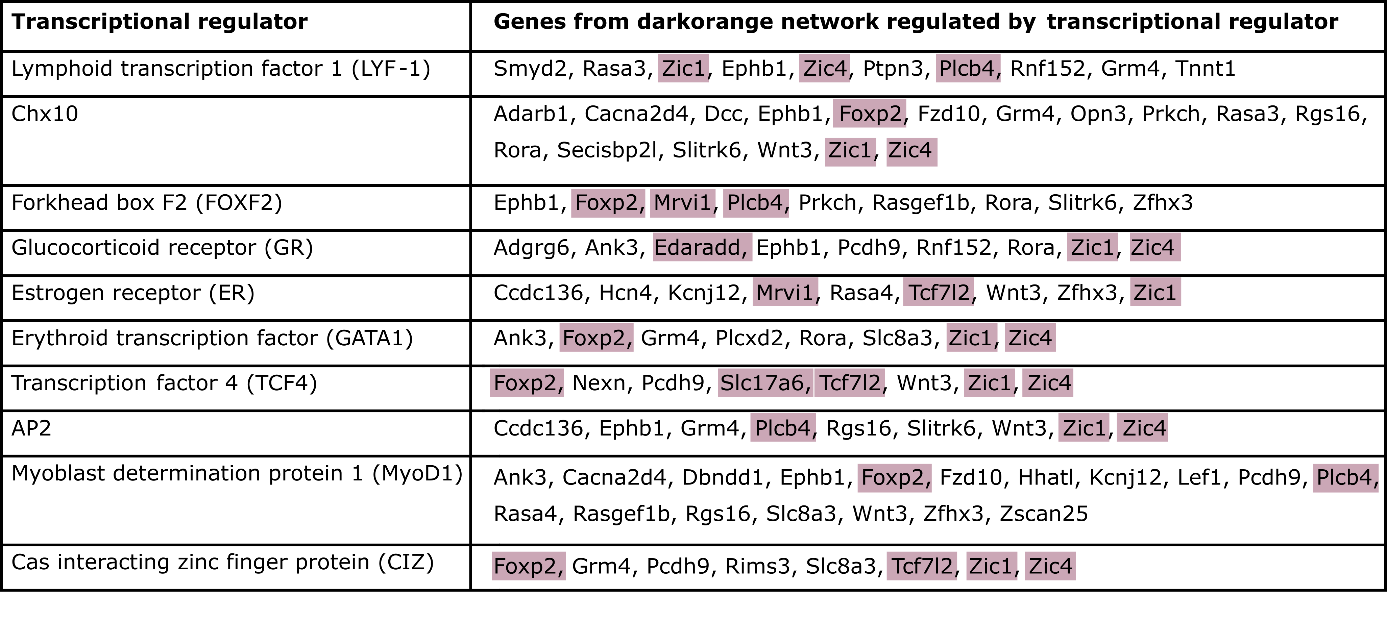
**

**Supplementary Figure 6. Enriched transcription factors of the darkorange network and their regulated genes**

A transcription factor enrichment analysis of the darkorange network revealed 10 enriched transcription factors. This figure shows the different enriched transcription factors and their targets from the darkorange network. These target genes from the darkorange network were later overlaid with different human psychiatric GWAS datasets and the dataset with hub genes from the darkorange network. The genes that are highlighted were found to have an overlap with any of the selected datasets.

**
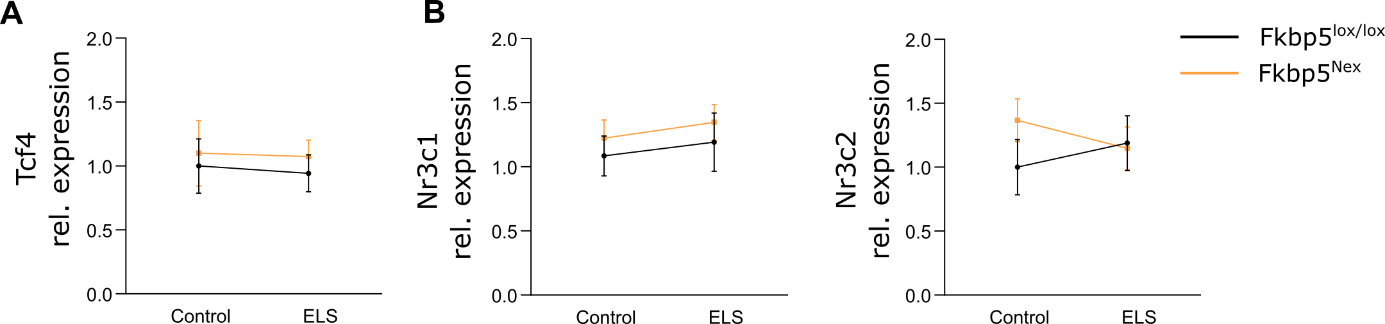
**

**Supplementary Figure 7. Expression of Tcf4 and corticosterone receptors in the hippocampus of ELS females**

(A) qPCR verified that the darkorange network hub gene TCF4 is not itself differentially expressed in females. (B) GR (Nr3c1) and MR (Nr3c2) are not differentially expressed following ELS in Fkbp5^Nex^ or Fkbp5^lox/lox^ mice. Each data point represents a distinct group of mice, and lines are used solely to enhance visual clarity. Error bars represent mean + S.E.M. All panels: 2-way ANOVA.

**
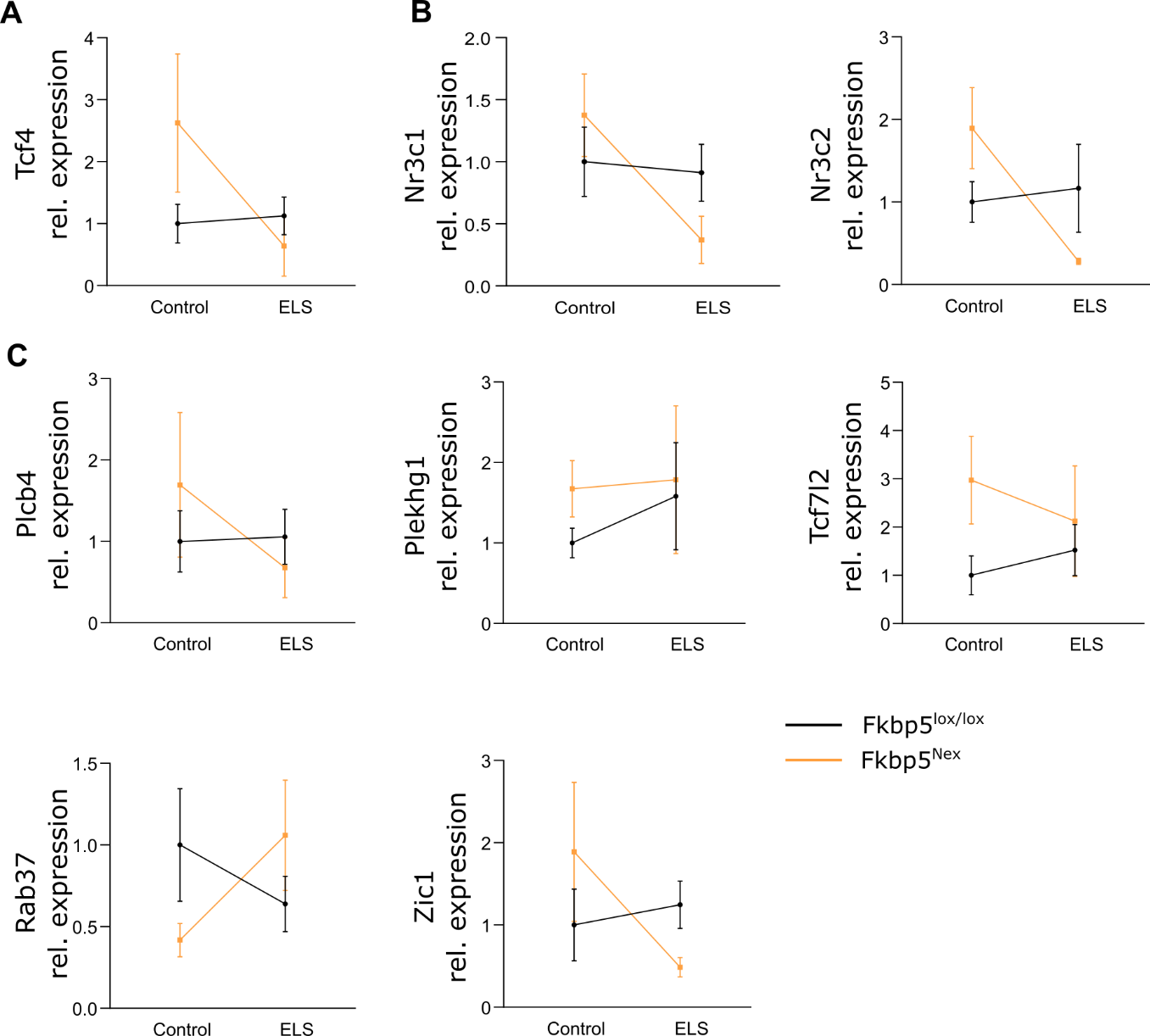
Supplementary Figure 8. Expression of Tcf4 target and hub genes in the hippocampus of ELS males**

(A) qPCR verified that the darkorange network hub gene TCF4 is not differentially expressed in males. (B) The corticosterone receptors GR (Nr3c1) and MR (Nr3c2) are not differentially expressed following ELS in Fkbp5^Nex^ or Fkbp5^lox/lox^ mice. (C) The five hubgenes of the darkorange network from the WGCNA analyses in females are not differentially expressed following ELS in Fkbp5^Nex^ or Fkbp5^lox/lox^ male mice. Each data point represents a distinct group of mice, and lines are used solely to enhance visual clarity. Error bars represent mean + S.E.M. All panels: 2-way ANOVA.

**
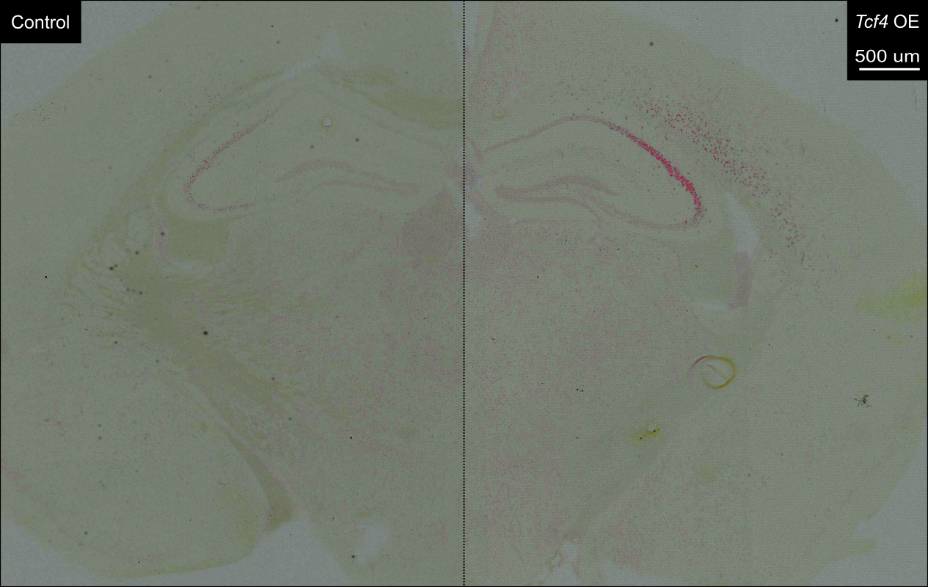
**

**Supplementary Figure 9. Validation of viral Tcf4 overexpression**

Example pictures of TCF4 expression under control conditions (left panel) and following hippocampal viral overexpression (right panel).

**
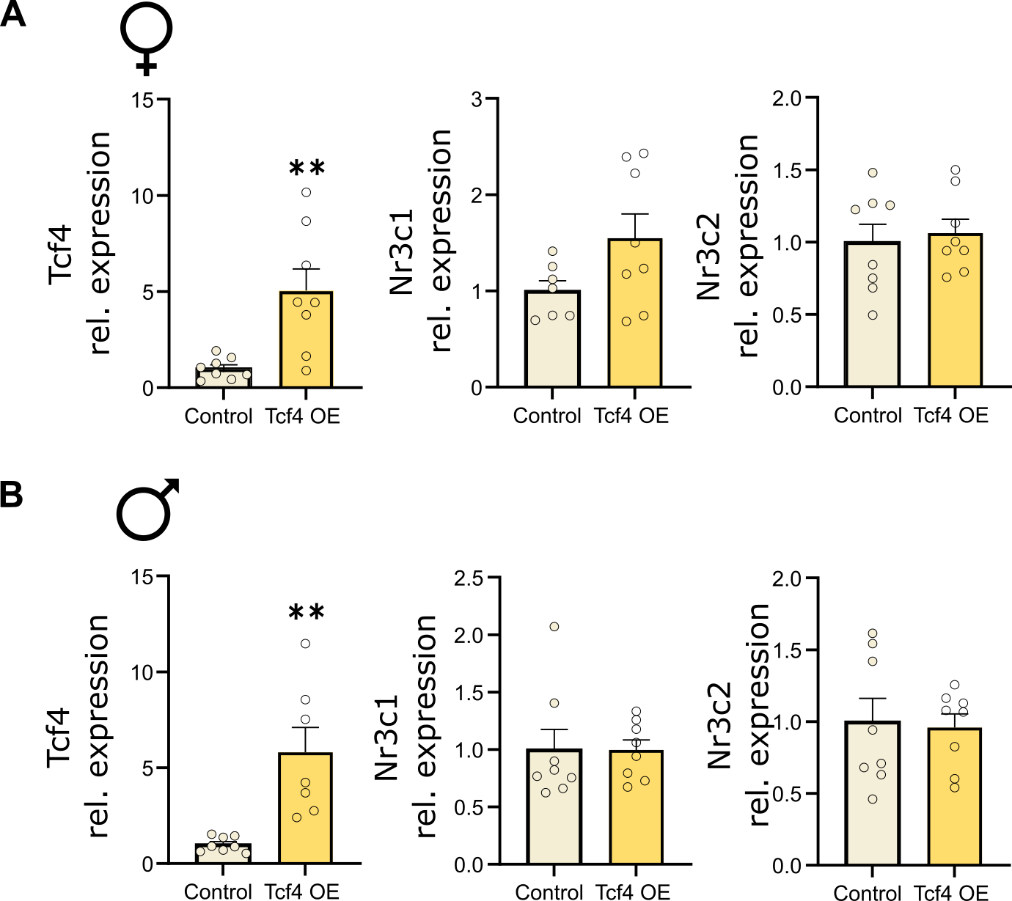
**

**Supplementary Figure 10. Tcf4 overexpression does not affect GR or MR expression in males and females in the hippocampus**

Tcf4 overexpression in females (A) or males (B) does not affect the expression of GR (Nr3c1) or MR (Nr3c2). n=8 per group. Error bars represent mean + S.E.M. All panels: 2-sided t-test. *** p < 0.001
