# Supplementary material for: FKBP51 in glutamatergic forebrain neurons promotes early life stress inoculation in female mice
Source: Nat Commun. 2025 Mar 14;16:2529. doi: 10.1038/s41467-025-57952-x (PMC11912546; doi:10.1038/s41467-025-57952-x)
Supplement: Supplementary file 2 — Reporting Summary [file 41467_2025_57952_MOESM2_ESM.pdf]

nature portfolio

Corresponding author(s): Mathias V. Schmidt
NCOMMS-24-19786-T

Last updated by author(s): Apr 5, 2024

# Reporting Summary

Nature Portfolio wishes to improve the reproducibility of the work that we publish. This form provides structure for consistency and transparency in reporting. For further information on Nature Portfolio policies, see our Editorial Policies and the Editorial Policy Checklist.

## Statistics

For all statistical analyses, confirm that the following items are present in the figure legend, table legend, main text, or Methods section.

| n/a | Confirmed | |
|---|---|---|
| ☐ | ☒ | The exact sample size (*n*) for each experimental group/condition, given as a discrete number and unit of measurement |
| ☐ | ☒ | A statement on whether measurements were taken from distinct samples or whether the same sample was measured repeatedly |
| ☐ | ☒ | The statistical test(s) used AND whether they are one- or two-sided<br>*Only common tests should be described solely by name; describe more complex techniques in the Methods section.* |
| ☒ | ☐ | A description of all covariates tested |
| ☐ | ☒ | A description of any assumptions or corrections, such as tests of normality and adjustment for multiple comparisons |
| ☐ | ☒ | A full description of the statistical parameters including central tendency (e.g. means) or other basic estimates (e.g. regression coefficient) AND variation (e.g. standard deviation) or associated estimates of uncertainty (e.g. confidence intervals) |
| ☐ | ☒ | For null hypothesis testing, the test statistic (e.g. *F*, *t*, *r*) with confidence intervals, effect sizes, degrees of freedom and *P* value noted<br>*Give P values as exact values whenever suitable.* |
| ☒ | ☐ | For Bayesian analysis, information on the choice of priors and Markov chain Monte Carlo settings |
| ☒ | ☐ | For hierarchical and complex designs, identification of the appropriate level for tests and full reporting of outcomes |
| ☒ | ☐ | Estimates of effect sizes (e.g. Cohen's *d*, Pearson's *r*), indicating how they were calculated |

*Our web collection on statistics for biologists contains articles on many of the points above.*

## Software and code

Policy information about availability of computer code

| | |
|---|---|
| Data collection | ANY-maze 6.18 |
| Data analysis | For analyses the following software was used: ANY-maze 6.18, ImageJ 1.54, GraphPad Prism 9, IMARIS, R (version 4.2.1), SPM12, Neurolucida, Hisat2 (version 2.0.5), FeatureCounts (version 1.5.0-p3), QuantStudio |

For manuscripts utilizing custom algorithms or software that are central to the research but not yet described in published literature, software must be made available to editors and reviewers. We strongly encourage code deposition in a community repository (e.g. GitHub). See the Nature Portfolio guidelines for submitting code & software for further information.

## Data

Policy information about availability of data

All manuscripts must include a data availability statement. This statement should provide the following information, where applicable:

- Accession codes, unique identifiers, or web links for publicly available datasets
- A description of any restrictions on data availability
- For clinical datasets or third party data, please ensure that the statement adheres to our policy

RNAseq data have been deposited in Gene Expression Omnibus under accession number GSE256468. All other data are included in the manuscript and/or SI Appendix.

## Research involving human participants, their data, or biological material

Policy information about studies with human participants or human data. See also policy information about sex, gender (identity/presentation), and sexual orientation and race, ethnicity and racism.

| | |
|---|---|
| Reporting on sex and gender | n.a. |
| Reporting on race, ethnicity, or other socially relevant groupings | n.a. |
| Population characteristics | n.a. |
| Recruitment | n.a. |
| Ethics oversight | n.a. |

Note that full information on the approval of the study protocol must also be provided in the manuscript.

# Field-specific reporting

Please select the one below that is the best fit for your research. If you are not sure, read the appropriate sections before making your selection.

☒ Life sciences ☐ Behavioural & social sciences ☐ Ecological, evolutionary & environmental sciences

For a reference copy of the document with all sections, see nature.com/documents/nr-reporting-summary-flat.pdf

# Life sciences study design

All studies must disclose on these points even when the disclosure is negative.

| | |
|---|---|
| Sample size | Given the exploratory nature of the analyses, no sample size was calculated a priori. |
| Data exclusions | Outliers were identified as values greater than 2 times the standard deviation (SD) from the mean (M) and excluded from analyses. |
| Replication | Independent replication datasets were not included. |
| Randomization | Mice were randomly assigned to experimental groups. |
| Blinding | During data collection and analysis, the investigators were blinded to group allocation. |

# Reporting for specific materials, systems and methods

We require information from authors about some types of materials, experimental systems and methods used in many studies. Here, indicate whether each material, system or method listed is relevant to your study. If you are not sure if a list item applies to your research, read the appropriate section before selecting a response.

## Materials & experimental systems

| n/a | Involved in the study | |
|---|---|---|
| ☒ | ☐ | Antibodies |
| ☒ | ☐ | Eukaryotic cell lines |
| ☒ | ☐ | Palaeontology and archaeology |
| ☐ | ☒ | Animals and other organisms |
| ☒ | ☐ | Clinical data |
| ☒ | ☐ | Dual use research of concern |
| ☒ | ☐ | Plants |

## Methods

| n/a | Involved in the study | |
|---|---|---|
| ☒ | ☐ | ChIP-seq |
| ☒ | ☐ | Flow cytometry |
| ☐ | ☒ | MRI-based neuroimaging |

# Animals and other research organisms

Policy information about studies involving animals; ARRIVE guidelines recommended for reporting animal research, and Sex and Gender in Research

| | |
|---|---|
| Laboratory animals | FKBP51-Nex or NEX-Cre mice at 5-8 months of age on a C57Bl/6n background. All animals were bred at the in-house breeding facility of the Max Plank Institute of Psychiatry in Martinsried, Munich, DE. |
| Wild animals | n.a. |
| Reporting on sex | Data were obtained in both male and female mice and reported accordingly. |
| Field-collected samples | n.a. |
| Ethics oversight | All experiments were in accordance with the European Communities Council Directive 2010/63/EU and were approved by the committee for the Care and Use of Laboratory animals of the Government of Upper Bavaria. |

Note that full information on the approval of the study protocol must also be provided in the manuscript.

# Plants

| | |
|---|---|
| Seed stocks | n.a. |
| Novel plant genotypes | n.a. |
| Authentication | n.a. |

# Magnetic resonance imaging

## Experimental design

| | |
|---|---|
| Design type | deformation based morphometry (DBM) |
| Design specifications | n.a. |
| Behavioral performance measures | n.a. |

## Acquisition

| | |
|---|---|
| Imaging type(s) | structural |
| Field strength | 9.4 |
| Sequence & imaging parameters | 3D gradient echo, cartesian k-space sampling, field of view 19.8 x 12.8 x 15.8 mm^3, matrix size 256 x 166 x 205, coronal read orientation, TE/TR/flip angle 6.253ms/34.1ms/10 degree |
| Area of acquisition | whole brain scan |

Diffusion MRI ☐ Used ☒ Not used

## Preprocessing

| | |
|---|---|
| Preprocessing software | SPM12 on Matlab R2022b, brain extraction based on tissue probability maps, SPM12 "old segment" function, isotropic smoothing kernel size = 5.7 x voxel size |
| Normalization | non-linear registration (DARTEL), flow fields transformed to jacobian deformation fields |
| Normalization template | study specific template, aligned with the Hikishima C57Bl6 mouse MRI template (Hikishima et al., Sci Rep 7, 85 (2017)) |
| Noise and artifact removal | spatial adaptive non-local means denoising filter (CAT12) |

Volume censoring | n.a.

## Statistical modeling & inference

Model type and settings: deformation based morphometry

Effect(s) tested: two-way ANOVA (early life stress x genotype and interaction if statistically significant), corrected for total brain volume

Specify type of analysis: ☒ Whole brain ☐ ROI-based ☐ Both

Statistic type for inference (See Eklund et al. 2016): cluster

Correction: FWE correction at the cluster level (pFWE,cluster < 0.05), with a cluster collection threshold of p < 0.005 uncorrected

## Models & analysis

| n/a | Involved in the study |
|---|---|
| ☒ | ☐ Functional and/or effective connectivity |
| ☒ | ☐ Graph analysis |
| ☒ | ☐ Multivariate modeling or predictive analysis |
